# A novel melting curve-based method for detecting *c-kit* mutations in acute myeloid leukemia

**DOI:** 10.3892/ol.2014.2128

**Published:** 2014-05-09

**Authors:** QUANYI LU, XIAO HUANG, HUAYING CHEN, XIAOMIN ZHAO

**Affiliations:** Department of Hematology, Zhongshan Hospital of Xiamen University, Xiamen, Fujian 361004, P.R. China

**Keywords:** *c-kit*, genetic mutation, melting curve, leukemia, mutation detection

## Abstract

The *c-kit* gene encodes a class III tyrosine kinase receptor. Specific somatic mutations in *c-kit* have been associated with acute myeloid leukemia (AML) and are markers of a poor prognosis in AML. Various methods have been used to detect the *c-kit* gene mutation; however, the suitability of these methods in the clinical management of AML remains unclear. The current study developed a novel method, using modified hybridization probes and melting curve analysis, for detecting *c-kit* mutations in exon 17. Dual-labeled self-quenched oligonucleotide probes containing two segments, labeled with carboxyrhodamine or hexachlorofluorescein, were designed to detect sequences around the D816 or N820/N822 mutation hot spots in exon 17 of *c-kit*. The exon 17 region of *c-kit* was amplified by polymerase chain reaction using control plasmids carrying wild-type or mutant sequences, or genomic DNA derived from AML patients. Melting curve analysis of the amplification products was performed using a self-quenched probe. The results showed that the detection sensitivity, assayed using mutation-positive control plasmids, was 10% for the N820G mutation and 5% for the six other mutations; N822K(A), N822K(G), D816V, D816Y, D816H and D816F. In addition, *c-kit* mutations were identified in six of the 12 samples from the core-binding factor (CBF)-AML patients. This demonstrates that the novel method developed in the present study, is simple, rapid, specific and highly sensitive, and may facilitate the diagnosis and treatment of CBF-AML.

## Introduction

Chromosomal translocations and gene mutations are common genetic abnormalities observed in leukemia patients (1). In total, ~50% of patients with acute myeloid leukemia (AML) carry a distinct chromosomal translocation, such as t(8;21) (q22;q22) or t(8;21), the latter of which ~10% of all AMLs exhibit and is considered to be an AML group that is associated with a favorable prognosis. The t(8;21)(q22;q22) and inv(16)(p13.1;q22)/t(16;16)(p13.1;q22) chromosomal alterations are the most common genetic abnormalities and give rise to the *AML1-ETO* and *CBFB-MYH11* fusion genes, respectively. As *AML1* encodes the α subunit of the core-binding factor (CBF) and *CBFB* encodes its β subunit, these two gene fusions interfere with normal CBF function. Therefore, AML with *AML1-ETO* or *CBFB-MYH11* is termed CBF-AML and accounts for 15% of AML cases worldwide (2,3).

The *c-kit* gene is located on chromosome 4q11–12 and encodes a 145-kDa type III receptor tyrosine kinase. *c-kit* has five extracellular immunoglobulin-like domains, a juxtamembrane domain and an intracellular kinase domain. *c-kit* mutations have been identified in ≥70% of gastrointestinal stromal tumors, ≥90% of mastocytosis and ~10% of germ cell tumors (4,5). In addition, *c-kit* mutations have been found in 12–25% of CBF-AML cases (6). It has also been reported that CBF-AML cases exhibiting a *c-kit* mutation are associated with a higher rate of relapse and a poor prognosis (7,8). Thus, the *c-kit* mutation may be a prognostic factor for CBF-AML.

Various methods have been used to detect *c-kit* mutations and one of the most common methods is the amplification refractory mutation system (9). However, its application is limited due to the requirement for high primer concentrations, its ability to only detect a small quantity of mutation sites and the complexity of the detection process. High-resolution melting analysis (10) detects DNA mutations based on the melting characteristics of the DNA molecules. It is an additional method that is relatively simple, however, it may be too sensitive as the ion concentrations in the samples may affect the results. Currently available hybridization probes (11) only detect mutations around the hot spot at D816 and, although frequently used at present, denaturing high-performance liquid chromatography combined with direct sequencing (12) requires the polymerase chain reaction (PCR) products to be post-processed, which may result in contamination. Furthermore, this method is complex and not applicable for mutation detection in clinical samples. Therefore, a simple, accurate and highly efficient method is required for detecting *c-kit* mutations.

Our previous study established a novel melting curve-based method for detecting gene mutations (13). In the present study, a unique probe arrangement was designed to establish a novel melting curve-based method for detecting *c-kit* mutations. The results demonstrated that this method detected the majority of mutations at the exon 17 hot spot. Furthermore, this method is advantageous due to its simplicity combined with its high sensitivity and specificity.

## Materials and methods

### Clinical samples

Bone marrow (2 ml) or peripheral blood (5 ml) samples were collected from 107 patients with leukemia at the Zhongshan Hospital of Xiamen University (Xiamen, China), between July 2008 and January 2010. All patients were diagnosed in accordance with the leukemia diagnostic standards (14), which was confirmed by morphological and immunophenotypic analyses of the bone marrow. Of the samples, 12 were from CBF-AML patients who were positive for *AML-ETO*. The patients provided written informed consent for the collection of the bone marrow and blood samples for the diagnostic and study purposes in accordance with the principles outlined in the Code of Ethics of the World Medical Association (Declaration of Helsinki). The experimental procedures were performed following the guidelines of the Xiamen University Medical Research Council and were approved by the ethics committee of the Zhongshan Hospital of Xiamen University.

### DNA extraction

Genomic DNA was extracted using a Qiagen genomic DNA extraction kit [Tiangen Biotech (Beijing) Co., Ltd., Beijing, China] within 24 h of the collection of the blood samples. The DNA concentration was measured using spectrophotometry (UV-2450/2550; Shimadzu Corp., Kyoto, Japan); the absorbance was measured at 260 nm and the DNA samples were diluted to a concentration of 10 ng/ml.

### Primer and probe design

**Figure 1:**
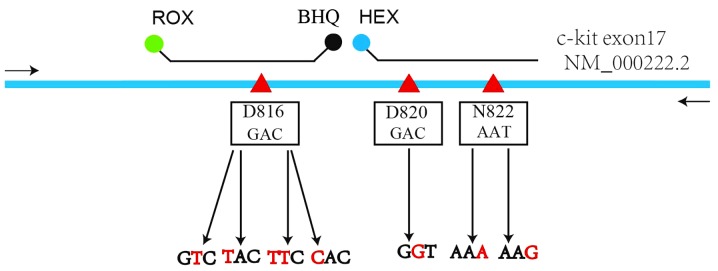
Schematic diagram of the common mutation sites in *c-kit* and the location of the primers and probes used in the present study. The red triangles represent the sites of common mutations and the wild-type *c-kit* sequences are shown in and under the boxes. The red capital letters beneath the boxes refer to the specific mutation sites. The primers (horizontal arrows) and probes (labeled with ROX, HEX and BHQ) are also indicated. ROX, carboxyrhodamine; HEX, hexachlorofluorescein; BHQ, black hole quencher.

Primer Premier v5.00 (Premier Biosoft, Palo Alto, CA, USA) and Tm Utility v1.3 (Sangon Biotech (Shanghai) Co., Ltd. (Shanghai, China) software packages were used to design the primers and probes. The primers and probes were synthesized by Sangon Biotech (Shanghai) Co., Ltd. The probes contained the following two segments: i) A self-quenched probe segment labeled with a carboxyrhodamine (ROX) fluorophore at its 5′ end (the first three basic groups were thiophosphorylated to prevent shearing of the fluorophore-carrying basic group by the DNA polymerase) and a black hole quencher (BHQ) at its 3′ end; and ii) a probe segment labeled with a hexachlorofluorescein (HEX) fluorophore at its 5′ end and a NH_2_ group at its 3′ end to prevent probe extension by the DNA polymerase (Fig. 1, Table I). There were three basic groups between the two sequences; therefore, the quenching group of the first sequence was able to function with the two fluorophores. This resulted in the formation of a single probe in the first sequence and the formation of a hybridization probe when combined with the second sequence. In the combined probe that contained the two segments, the sequence of the first segment was designed to detect mutations around D816, and the sequence of the second segment was designed to detect mutations at N820 and N822. The hybridization of the probe to the target sequence that contained sequences around D816 alone, N820/N822 alone, or D816 and N820/N822 together enabled the signal from ROX alone, HEX alone, or ROX and HEX together to be detected. Furthermore, the melting curve analysis indicated the presence of unique sequences (a single peak, which was unique to the wild-type (WT) or mutant sequence) or mixtures of the sequences (multiple peaks, each corresponding to the WT or mutant sequence).

### Construction of mutation-positive plasmids

Using genomic DNA from 293T human embryonic kidney cells as the template, mutation-positive control plasmids were constructed using the overlap extension PCR method (15,16). The plasmids contained the following *c-kit* WT or mutant sequences: D816WT, D816V, D816Y, D816H, D816F, N822K(A), N822K(G) and N820G. D816WT contained the WT sequence, while in D816V, the GAC codon for amino acid 816 was mutated to GTC, resulting in a D (aspartic acid) to V (valine) change. The relevant plasmid sequences are listed in Table II.

### PCR amplification and mutation detection

The PCR reactions contained 1× sequence-specific primer buffer [67 mM Tris-HCl, 16.6 mM (NH_4_)_2_SO_4_, 6.7 μM EDTA and 0.085 mg/ml bovine serum albumin], 4 mM Mg^2+^, 0.2 mM dNTPs, 1 pmol upstream primer, 10 pmol downstream primer, 2 pmol D816-ROX probe, 2 pmol N822-HEX probe, 1 unit of Taq HS, 5 μl of template DNA and ddH_2_O in a final volume of 25 μl.

Amplification was conducted using a Gene-pro Gene Amplifier (Bioer Biotechnology Co., Ltd., Hangzhou, China) with 50 cycles of 95°C for 20 sec, 52°C for 30 sec and 72°C for 30 sec. The melting curves were analyzed using a CFX96 Real-Time PCR detection system (Bio-Rad, Hercules, CA, USA) and measurement of fluorescence (HEX and ROX channels) at 0.5°C increments was performed between 35 and 80°C.

### Sensitivity testing

The mutation-positive control plasmids were diluted to 2×10^3^ copies/μl. The WT and control plasmids were used as templates to produce mixtures with 50, 25, 10, 5 and 1% of the plasmids that contained the individual mutations. The plasmid mixtures were used as templates for amplification and mutation detection. The samples were tested in duplicate, together with a WT-positive control and template-free negative control.

### Sample detection and sequencing

In total, 5-μl aliquots of DNA samples from patients were used for amplification and melting curve analysis. In addition, the 12 PCR products from the CBF-AML patients were sequenced using a commercial sequencing service (Major Biosystem Co., Ltd., Shanghai, China). The results of the sequencing analysis of the patient DNA samples were compared with those of the mutation-positive control plasmids.

## Results

### Sensitivity of the mutation detection system

**Figure 2:**
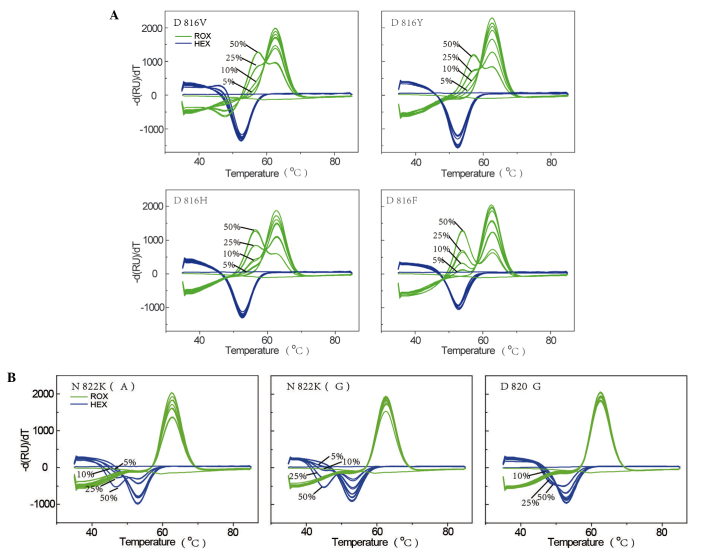
Evaluation of the sensitivity of the method for detecting hot spot mutations in the *c-kit* gene. The method detected all mutations, with the exception of D820(G), at a sensitivity of 5% mutation in a mixture of wild-type and mutant sequences. ROX, carboxyrhodamine; HEX, hexachlorofluorescein.

To test the sensitivity of the novel system, the plasmid mixtures containing the plasmid with the WT *c-kit* sequence and each of the seven plasmids carrying *c-kit* mutations were examined. The mutations included four D816 mutations (D816V, D816Y, D816H, and D816F), two N822 mutations [N822K(A) and N822K(G)], and a N820 mutation (N820G). The results of the sensitivity analysis are shown in Fig. 2. For the four D816 mutations, the signal from the ROX channel for the WT plasmid exhibited only one melting peak (at ~62.5°C), whereas the 50, 25, 10 and 5% plasmid mixtures exhibited double peaks that clearly differed from that of the WT plasmid. Double peaks were not evident for the 1% mixture, indicating that the detection sensitivity for the four D816 mutations was ~5%. For the remaining mutations, the signal from the HEX channel for the WT plasmid exhibited only one melting peak (at ~52.5°C), while the mixed plasmids exhibited double peaks. The detection sensitivity for N822K(A) and N822K(G) was 5%, while that of N820G was 10%.

### Melting curve and sequencing analyses of CBF-AML samples

**Figure 3:**
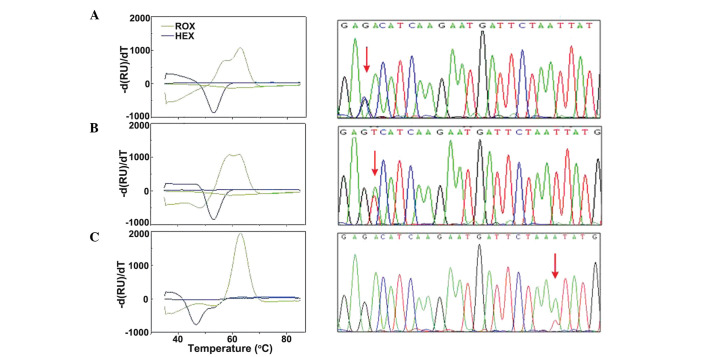
Melting curves and sequencing analysis of mutation-positive samples. The melting curves (left panel) are shown and divided into two parts; the ROX and HEX signals. The appearance of a double peak in the melting curve is indicative of the presence of the wild-type and mutant sequences, while a single abnormal peak is indicative of the presence of a mutation. Five abnormal melting curves, corresponding to the five mutations analyzed in the present study are shown. The sequencing results of the *c-kit* gene for samples from *AML-ETO*-positive patients are shown on the right. The red arrows indicate the mutated bases. Data are presented for the (A) D816H, (B) D816V and (C) N822 mutations. ROX, carboxyrhodamine; HEX, hexachlorofluorescein.

The results of the melting curve and sequencing analyses for the 12 CBF-AML patient-derived samples are shown in Fig. 3. The ROX signal identified four samples for which the melting curve was different from that of the WT sequence, indicating a mutation at D816. Three samples exhibited a single peak at 57.5°C, however, they were clearly different from the WT peak at 62°C. The final sample that was different exhibited a melting peak at 62°C (WT) and an additional peak at 56.5°C.

The HEX signal identified one sample with an abnormal HEX melting peak, with a melting peak at 54°C (WT) and an additional peak at 46°C, indicating the presence of a mutation at N820 or N822. Sequencing analysis of the 12 samples supported the melting curve data. Among the five *c-kit* mutation-positive samples, three different mutations were identified: A D816H mutation (one sample), a D816V mutation (three samples) and a N822K(A) mutation (one sample; Fig. 3). No mutations were detected in the remaining six CBF-AML cases.

### Melting curve analysis of non-CBF-AML samples

**Figure 4:**
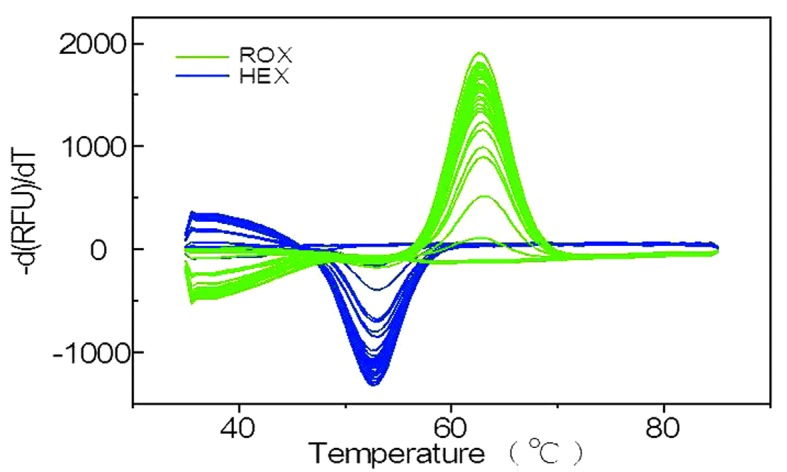
Results of the mutation detection assay in samples from the non-core-binding factor acute myeloid leukemia patients. ROX and HEX signal detection indicated that all samples corresponded with the wild-type *c-kit* sequences. ROX, carboxyrhodamine; HEX, hexachlorofluorescein.

To assess the *c-kit* mutation rate in samples from patients with other types of leukemia, the novel method was used to analyze 95 non-CBF-AML samples, including 58 AML (negative for *AML1-ETO* and *CBFB-MYH11*), 25 acute lymphoblastic leukemia, 10 chronic myelocytic leukemia and two chronic eosinophilic leukemia samples. Representative data for the 31 samples are shown in Fig. 4. Two samples exhibited no amplification signals, while the remaining 29 ROX and HEX signals exhibited single melting peaks, indicating that the signals were negative for *c-kit* mutations.

## Discussion

Aberrant *c-kit* in t(8;21) AML has been reported in the extracellular domain (encoded in exon 8), the juxtamembrane domain (encoded in exons 10 and 11) and the A-loop domain with tyrosine kinase activity (encoded in exon 17). Certain previous studies reported that the D816V mutation (in exon 17) confers increased tumor growth and antiapoptotic potential compared with mutations in the extracellular or juxtamembrane domains (17,18). Therefore, it was hypothesized that the development of a highly sensitive method for detecting *c-kit* mutations at exon 17 is required and may facilitate the appropriate management of AML.

The current study modified a previously described hybridization probe technique (13), where a single quencher was used to quench two fluorophores on the probe. In this modified probe method, the anterior segment of the probe was an independent self-quenching probe labeled with a fluorophore (ROX) at its 5′ end and a BHQ at its 3′ end. In addition, the first three basic groups at the 5′ end were thiophosphorylated to prevent the shearing effects that are caused by the excision step of DNA polymerase on the fluorophore-carrying basic group. In the annealing step, the probe hybridized to the amplification product and product-specific unique sequence information was obtained from the melting curve analysis. The posterior segment of the probe was an oligonucleotide labeled with a different fluorophore (HEX) at its 5′ end and the sequence of this oligonucleotide allowed for hybridization with the front half of the probe, enabling the hybridization of the amplification products during annealing. The melting curve analysis of the probe-covered regions directly reflected the sequence of the region. This modified probe is advantageous as it provides sequence information by overlapping the two segments of the probe, whereas the original hybridization probe only reveals sequence information in the region that is overlapped by the fluorescent probe. However, as the region that is covered by two segments of the probe is long, the detection of one self-quenched or molecular probe may not provide sufficiently high fluorescence signals or may fail to detect mutations due to reduced sensitivity.

It is known that WT DNA may interfere with the detection of mutant DNA. Therefore, it is important to analyze sensitivity. The gold standard sensitivity for *c-kit* mutation detection has been set at 20% (11). The method used in the current study exceeded this threshold for sensitivity for all the mutations analyzed; the sensitivity was 10% for N820G and 5% for the other six mutations tested.

In the present study, *c-kit* mutations were identified in six of the 12 *AML-ETO*-positive samples, yielding a positivity rate (50%) comparable with those previously reported; 12.8–46.8% (19–21). Furthermore, to evaluate *c-kit* mutations in non-CBF-AML cases, *c-kit* mutations were also analyzed in 95 samples obtained from non-CBF-AML patients. As predicted, no *c-kit* mutations were identified, which indicates that the *c-kit* mutation is rare in non-CBF-AML cases.

In conclusion, the method described in the present study is simple and rapid, and exhibits high sensitivity and specificity. This modified probe method may facilitate the classification and individual treatment of patients with CBF-AML.

## Figures and Tables

**Table I:** Primer and probe seqences.

A, Primers	

Description	Sequence (5′ to 3′)
d-Kit17-F1	ACAGAGACTTGGCAGCCAGAA
d-Kit17-R	TTGCAGGACTGTCAAGCAGAG

B, Probes	

Description	Sequence (5′ to 3′)

D816-ROX^a^	ROX-TGGTCTAGCCAGAGaCATCAA-BHQ
N822-HEX^a^	HEX-TGATTCTAATTATGTGGTTAAA-NH_2_

a The first three bases were modified with thiophosphorylation at the 5′ end.

ROX, carboxyrhodamine; BHQ, black hole quencher; HEX, hexachlorofluorescein.

**Table II:** Sequences of the different plasmids.

Mutation	Sequence
D816WT	…gtgattttggtctagccaga**gac**atcaagaatgattctaattatgtggttaaa…
D816V	…Gtgattttggtctagccaga**gTc**atcaagaatgattctaattatgtggttaaa…
D816Y	…gtgattttggtctagccaga**Tac**atcaagaatgattctaattatgtggttaaa…
D816H	…gtgattttggtctagccaga**Cac**atcaagaatgattctaattatgtggttaaa…
D816F	…gtgattttggtctagccaga**TTc**atcaagaatgattctaattatgtggttaaa…
N820G	…gtgattttggtctagccagagacatcaagaat**gGt**tctaattatgtggttaaa…
N822K(A)	…gtgattttggtctagccagagacatcaagaatgattct**aaA**tatgtggttaaa…
N822K(G)	…gtgattttggtctagccagagacatcaagaatgattct**aaG**tatgtggttaaa…

Emboldend bases, mutation sites; capitalized bases, specific base mutation.
